# Abortion and Fulminant Idiopathic Intracranial Hypertension

**DOI:** 10.7759/cureus.13501

**Published:** 2021-02-23

**Authors:** Jayachandran Selvaraj, Vamsidhar Veeranki, Sai Yasaswini Kommaraju, Pradeep Ravi, Subashini Kaliaperumal

**Affiliations:** 1 General Internal Medicine, Jawaharlal Institute of Postgraduate Medical Education and Research, Pondicherry, IND; 2 General Medicine, Jawaharlal Institute of Postgraduate Medical Education and Research, Pondicherry, IND; 3 Ophthalmology, Jawaharlal Institute of Postgraduate Medical Education and Research, Pondicherry, IND

**Keywords:** idiopathic intracranial hypertension, fulminant, visual loss, anemia

## Abstract

Idiopathic intracranial hypertension (IIH) causes visual involvement secondary to papilledema but rarely presents with acute gross diminution of vision. Anemia is associated with IIH. Acute hemorrhage-related anemia causing severe sudden onset bilateral visual loss due to IIH has not been previously reported. A 28-year-old female attempted the first-trimester abortion by self-administration of oral drugs. She presented with bleeding *per vaginum*, followed by bilateral visual loss. Symptoms pertaining to intracranial hypertension were mild. Examination revealed pallor, normal hemodynamic parameters, bilaterally dilated pupils, bilateral lateral rectus palsy, and only perception of light in both eyes. Lumbar puncture demonstrated high pressures; neuroimaging was noncontributory. Blood transfusion and supportive therapy in the form of acetazolamide and pulse methylprednisolone improved her vision in the right eye to six of 24; optic nerve sheath fenestration was performed in the left eye. During follow-up, her vision improved to six of 24 (right) and two of 60 (left), respectively. The IIH can present with severe acute onset bilateral visual loss even if features of raised intracranial pressure are minimal or absent. Immediate correction of anemia and supportive measures may significantly improve visual outcomes in fulminant IIH without the necessity of surgery.

## Introduction

Idiopathic intracranial hypertension (IIH) is associated with raised intracranial pressure (ICP) and visual involvement [1]. Vision involvement in IIH is secondary to papilledema and generally affects the peripheral field of vision [1]. Fulminant IIH (<3% of cases) causes severe vision loss within four weeks of onset, and the vision loss progresses over days, which has commonly been described in obese women in the reproductive age group [2]. We report a young non-obese woman with bleeding-related anemia following an abortion, after which she presented with fulminant IIH-related bilateral sudden onset loss of vision, albeit with only minimal symptoms of raised ICP.

## Case presentation

A 28-year-old female presented with amenorrhea for one and a half months and bleeding per vaginum for five days. She gave the history of attempting the first-trimester abortion by the administration of over-the-counter drugs: one tablet of mifepristone 200mg and four tablets of misoprostol 200mcg. Two days after the onset of bleeding, she had blurring of vision bilaterally, mild occipital headache, and two to three episodes of vomiting. The visual loss progressively worsened. There was no prior head trauma, neck pain, fever, or history to suggest sleep apnea.

**Figure 1:**
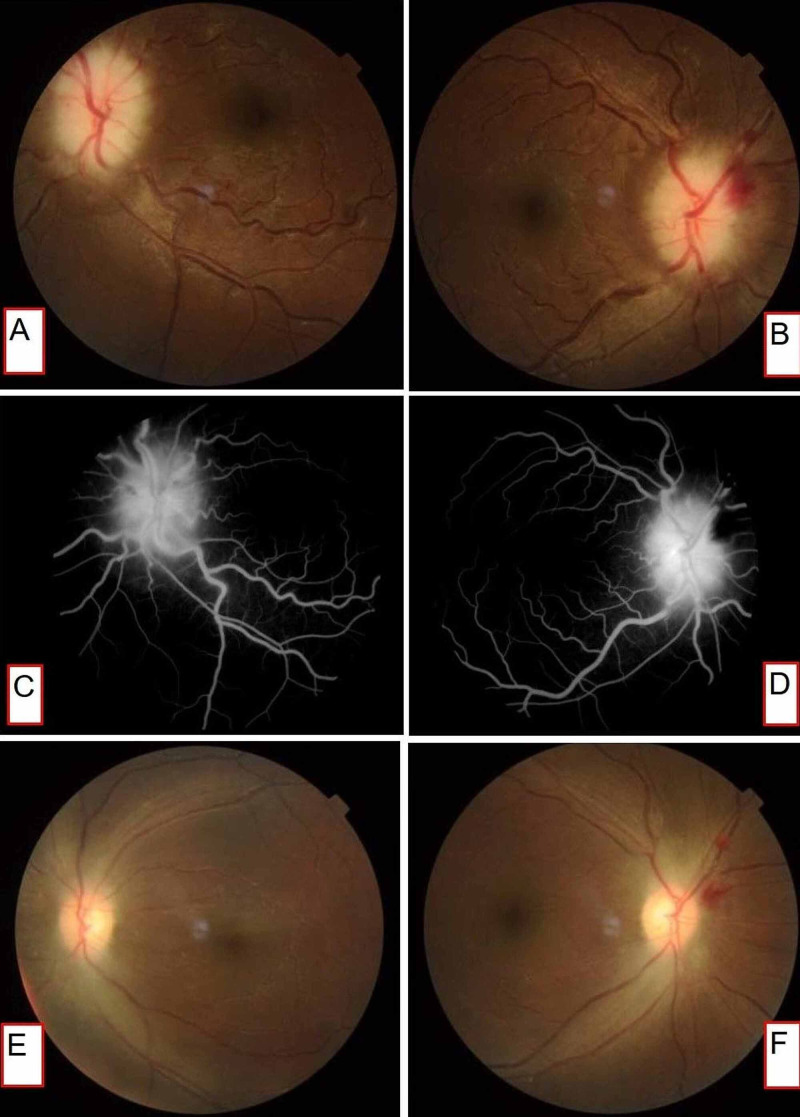
Fundi and fluorescein angiography (A and B) Fundus photograph at admission: elevated and diffusely blurred disc margins are noted along with tortuous vessels. Peripapillary hemorrhages are seen in the temporal aspect of the left eye. (C and D) FFA photograph at admission: fluorescein dye leak at the disc suggestive of true papilledema. No vascular filling defects were noted. (E and F) Fundus photograph at discharge: significant improvement in papilledema can be seen.

Examination revealed pallor, normal hemodynamic parameters, body mass index (BMI) of 23.7kg/m^2^ (60kg/1.5m^2^). A hemic murmur was present in the pulmonary area. Bilaterally dilated pupils and bilateral lateral rectus palsy were present. Visual acuity showed the perception of light (PL) in the right eye, and no PL in the left eye. Fundus examination revealed blurred margins with pale disc edema in both eyes (Figures 1A, 1B). There was no neck stiffness. A gynecology consult and examination revealed that she had had a complete abortion, and evacuation was not indicated.

She was initially admitted under ophthalmology services with a diagnosis of Anterior Ischemic Optic Neuropathy (AION), which was attributed to severe anemia, and a medicine consult was obtained. Because of associated headache, vomiting, and bilateral lateral rectus palsy, raised ICP was suspected as the cause of papilledema and vision loss. There was no vascular occlusion on fluorescein fundus angiography (FFA) (Figures 1C, 1D). Non-contrast CT on day 2 and MRI/venogram (MRI/V) on day 3 were normal. Lumbar puncture (LP) on day 3 revealed an opening pressure of 840mmH_2_O (Table 1). Cerebrospinal fluid (CSF) analysis for infective and malignant causes of raised ICP was noncontributory. Hemoglobin (Hb) 4.9g/dL and microcytic hypochromic red blood cells, with otherwise normal complete blood counts, were observed. She received two red blood cell (RBC) transfusions on day 3 and two more on day 5, intending to maintain a hemoglobin of 10g/dL. On day 5 her vision in the right eye improved to two of 60. Her thyroid and renal profile were normal.

**Table 1:** CSF pressures and visual acuity during the hospital stay CSF: cerebrospinal fluid; PL: perception of light; HMCF: hand movements close to the face

Day	CSF Pressure (mmH_2_O)	Visual Acuity - Right Eye	Visual Acuity - Left Eye	Remarks
Day 3	840	PL+	PL -	30mL CSF tapped
Day 4		PL+	PL-	
Day 5	400	2/60	HMCF	30mL CSF tapped
Day 8	270	6/60	HMCF	30mL CSF tapped
Day 10	250			20mL CSF tapped
Day 14	200			30mL CSF tapped
Day 20 (discharge)	-	6/24	HMCF	-

Methylprednisolone (1g/day x 3 days) was initiated on day 3 in view of vision-impairing IIH. Mannitol 1gm/kg stat followed by 0.5gm/kg Q8H, acetazolamide, diclofenac (for pain relief), and ranitidine were also administered [3,4]. Headache and vomiting had entirely subsided after the first LP. A significant improvement in her right eye vision was noted within 24 hours of the first LP (Table 1). In the left eye, only hand movements close to the face (HMCF) were detected, so repeated therapeutic LP was done since she was unwilling for a lumbar drain (Table 1). Her right eye vision improved with repeated LP, but due to persistent poor vision in the left eye, optic nerve sheath fenestration (ONSF) was performed on day 16. Papilledema and visual acuity (right) also improved significantly on day 20 (Figures 1E, 1F). She was not willing for further surgical procedures. Oral prednisolone 1mg/kg was continued for two weeks, and a taper was planned. Her hemoglobin improved to 10.6g/dL at the time of discharge.

Her BMI increased to 28.5kg/m^2^; steroids were tapered gradually over four weeks and discontinued, and acetazolamide was gradually tapered to 500mg/day [4]. There was no recurrence of symptoms. At two months, vision improved to six of 24 (right) and two of 60 (left), respectively. Oral hematinics were continued for three months.

## Discussion

Idiopathic intracranial hypertension is a subset of patients with pseudotumor cerebri syndrome (PTCS) with an increase in ICP and normal CSF contents in the absence of an intracranial mass, hydrocephalus, or another identifiable cause [5]. Anemia is a cause of secondary pseudotumor cerebri syndrome [5]. A diagnosis of PTCS requires evidence of raised ICP (papilledema, cranial nerve deficits, normal neuroimaging, CSF pressure >250mmH_2_O in adults or >280mmH_2_O in children with normal CSF composition) [5]. Typically seen in young and obese females, symptoms of raised ICP are more prominent than that of visual complaints. Transient visual obscurations (TVO), noted in 68-85% of patients, are the most common visual symptoms in IIH [6]. Although perimetry detects visual field defects in 96% of cases, symptomatic visual field defects are seen only in 50% [7]. Visual acuity, on the other hand, is affected in only < 5% of patients with IIH [8]. Severe visual loss within four weeks of symptom onset is referred to as fulminant IIH, as seen in our patient [1]. Less than 3% of patients develop fulminant IIH [1].

In the largest series of 16 cases of fulminant IIH, the mean duration of decline in visual acuity following the onset of symptoms was 16 days (7-28 days) [1]. All patients had a severe headache, and most of them had recurrent vomiting in contrast to our patient, who had only minimal symptoms of raised ICP. The mean opening CSF pressure in that series was 541mmH_2_O in contrast to our patient’s 840mmH_2_O. The minimal symptoms despite such a high ICP could not be explained in our patient. Severe loss of vision within 24 hours of symptom onset and prominent visual complaints rather than features of raised ICP were characteristic in our patient. There are a few reports of a sudden loss of vision due to IIH, albeit with long-standing papilledema due to IIH [9]. One patient had a central retinal artery occlusion, and the other had a subretinal membrane associated with sudden vision loss, neither of which was seen in our patient. Acutely, autoimmune hemolytic anemia and parvovirus 19-related hemolytic anemia have been reported to cause IIH, but vision loss has not been described [10]. Though menorrhagia is known to cause chronic anemia-related IIH, acute hemorrhage following an abortion has never been previously reported to cause IIH-related visual loss [11]. Prominent visual symptoms are considered red flags while diagnosing IIH [5].

While severe iron deficiency anemia often occurs with raised ICP, its role is not established about either causation or association. There is a school of thought that mandates anemia be excluded before labeling a case of raised ICP as IIH. However, there is no consensus on this viewpoint [11]. Mollan et al. described eight patients with anemia and raised ICP, with the maximum opening CSF pressure of 520mmH_2_O and mean hemoglobin of 7.9g/dL. All but one improved with correction of anemia alone; a ventriculoperitoneal shunt was performed for the patient without symptom relief [12]. In a retrospective study from China, anemia with IIH (22/153) was associated with shorter disease duration and better visual outcome following correction of anemia [13]. In another case-control study, no significant difference was observed between anemic patients with IIH and matched controls with other ophthalmic diagnoses [14]. Our patient also required additional treatment with carbonic anhydrase inhibitors, steroids, and serial lumbar punctures as the symptoms did not alleviate with the initial blood transfusion. Steroids were initiated empirically as a temporizing therapy. ONSF was performed since the improvement in acuity of the left eye was minimal. We initially thought that the early improvement with RBCs, LP, and steroids would lead to a marked improvement in her visual acuity as well since her raised ICP features disappeared within 24 hours, and subsequent LPs showed falling CSF pressures. Hence the ONSF was delayed, which we probably erred in retrospect. There is no consensus regarding the target hemoglobin for anemia-associated IIH and the indications to consider diversion procedures for raised ICP in such cases. Although anemia is known to precipitate AION and fundus finding of pale disc edema was suggestive, it is unlikely to be the etiology in our case, as bilateral vision loss, symptoms of raised ICP, diffuse optic disc involvement, peripapillary hemorrhages, and lateral rectus palsy were suggestive of intracranial hypertension [11]. The residual visual defects in her could be due to poor prognostic factors like severe vision loss at presentation and the delayed surgery.

## Conclusions

The IIH can rarely be precipitated by acute blood loss-related anemia and can present with severe acute onset bilateral visual loss even if features of raised ICP are minimal or absent. Immediate correction of anemia, prompt surgery, and other supportive measures to reduce ICP may significantly improve visual outcomes.
